# The central regulatory circuit in the gene network controlling the morphogenesis of Drosophila mechanoreceptors: an in silico analysis

**DOI:** 10.18699/VJGB-23-87

**Published:** 2023-12

**Authors:** T.A. Bukharina, V.P. Golubyatnikov, D.P. Furman

**Affiliations:** Institute of Cytology and Genetics of the Siberian Branch of the Russian Academy of Sciences, Novosibirsk, Russia Novosibirsk State University, Novosibirsk, Russia; Sobolev Institute of Mathematics of the Siberian Branch of the Russian Academy of Sciences, Novosibirsk, Russia; Institute of Cytology and Genetics of the Siberian Branch of the Russian Academy of Sciences, Novosibirsk, Russia Novosibirsk State University, Novosibirsk, Russia

**Keywords:** central regulatory circuit, gene network, mathematical model, computer modeling, drosophila, achaetescute complex, mutations, центральный регуляторный контур, генная сеть, математическая модель, компьютерное моделирование, дрозофила, achaete-scute комплекс, мутации

## Abstract

Identification of the mechanisms underlying the genetic control of spatial structure formation is among the relevant tasks of developmental biology. Both experimental and theoretical approaches and methods are used for this purpose, including gene network methodology, as well as mathematical and computer modeling. Reconstruction and analysis of the gene networks that provide the formation of traits allow us to integrate the existing experimental data and to identify the key links and intra-network connections that ensure the function of networks. Mathematical and computer modeling is used to obtain the dynamic characteristics of the studied systems and to predict their state and behavior. An example of the spatial morphological structure is the Drosophila bristle pattern with a strictly defined arrangement of its components – mechanoreceptors (external sensory organs) – on the head and body. The mechanoreceptor develops from a single sensory organ parental cell (SOPC), which is isolated from the ectoderm cells of the imaginal disk. It is distinguished from its surroundings by the highest content of proneural proteins (ASC), the products of the achaete-scute proneural gene complex (AS-C). The SOPC status is determined by the gene network we previously reconstructed and the AS-C is the key component of this network. AS-C activity is controlled by its subnetwork – the central regulatory circuit (CRC) comprising seven genes: AS-C, hairy, senseless (sens), charlatan (chn), scratch (scrt), phyllopod (phyl), and extramacrochaete (emc), as well as their respective proteins. In addition, the CRC includes the accessory proteins Daughterless (DA), Groucho (GRO), Ubiquitin (UB), and Seven-in-absentia (SINA). The paper describes the results of computer modeling of different CRC operation modes. As is shown, a cell is determined as an SOPC when the ASC content increases approximately 2.5-fold relative to the level in the surrounding cells. The hierarchy of the effects of mutations in the CRC genes on the dynamics of ASC protein accumulation is clarified. AS-C as the main CRC component is the most significant. The mutations that decrease the ASC content by more than 40 % lead to the prohibition of SOPC segregation.

## Abstract (ru)

Выявление механизмов генетического контроля формирования пространственных структур остается одной из актуальных задач биологии развития. Для ее решения используются как экспериментальные, так и теоретические подходы и методы, в том числе методология генных сетей, а также методы математического и компьютерного моделирования. Реконструкция и анализ генных сетей, обеспечивающих становление признака, позволяют интегрировать существующие экспериментальные данные, выявить ключевые звенья и внутрисетевые связи, обеспечивающие функционирование сетей. Для получения динамических характеристик исследуемых систем, предсказания их состояния и поведения привлекаются методы математического и компьютерного моделирования. Одним из примеров пространственной морфологической структуры является щетиночный рисунок дрозофилы со строго определенным расположением на голове и теле мухи его составляющих – механорецепторов (внешних сенсорных органов). Механорецептор развивается из единственной родительской клетки (РКСО), которая выделяется из клеток эктодермы имагинального диска. Ее отличает от окружения наибольшее содержание пронейральных белков (ASC) – продуктов комплекса пронейральных генов achaete-scute (AS-C). Статус РКСО обеспечивается реконструированной нами ранее генной сетью, ключевым объектом которой является комплекс генов AS-C. Контроль активности комплекса осуществляется ее подсетью – центральным регуляторным контуром в составе семи генов (AS-C, hairy, senseless (sens), charlatan (chn), scratch (scrt), phyllopod (phyl), extramacrochaete (emc)) и одноименных белков. Кроме того, в состав центрального регуляторного контура входят вспомогательные белки Daughterless (DA), Groucho (GRO), Ubiquitin (UB) и Seven-in-absentia (SINA). В работе приведены результаты компьютерного моделирования различных режимов функционирования контура. Показано, что клетка детерминируется как РКСО при повышении содержания ASC примерно в два с половиной раза относительно уровня в клетках окружения. Выявлена иерархия влияния мутаций в генах контура на динамику накопления белков ASC. Наиболее значим главный компонент центрального регуляторного контура – AS-C. Мутации, снижающие содержание ASC более чем на 40 %, приводят к запрету выделения родительской клетки сенсорного органа.

## Introduction

The current views on the control of biological processes, including cell differentiation, growth and development of organisms, and construction of spatial structures, are united in the concept of gene networks. According to this concept, gene networks (GNs) are the molecular genetic systems that provide the formation of all phenotypic characteristics of organisms (molecular, biochemical, structural, morphological, ethological, physiological, cognitive, and so on) based on the information coded for in their genomes. Kolchanov et al. (2013) define GNs as the groups of concertedly operating genes that interact with one another via both their primary products (RNAs and proteins) and the diverse metabolites and other secondary products of GN operation.

The GNs are reconstructed based on the analysis of experimental data, which gives both the most comprehensive and systematized description of a considered biological system or a process (Schlitt et al., 2003; Zhu et al., 2007; Emmert- Streib, Glazko, 2011; Chasman et al., 2016). An important feature of the GNs is regulatory circuits, which ensure their correct function and implementation of the program that forms a phenotypic trait.

Mathematical and computer modeling makes it possible to acquire the most comprehensive insight into the GN arrangement and behavior and is widely used to clarify the structure–function organization of GNs, architecture of their inner links, detection of the key elements and modules, and patterns of their operation and evolution.

The GNs “Neurogenesis:prepattern”, “Neurogenesis:determination”, and “Neurogenesis:asymmetric division”, which we have earlier reconstructed are examples of the networks responsible for the development of ordered structures during ontogenesis. Together, these GNs provide a definite composition of mechanoreceptors (sensory organs of the peripheral nervous system) on the head and body of drosophila (Furman, Bukharina, 2022). Analysis of these networks has elicited the most important connecting link that controls their operation, namely, central regulatory circuit (CRC). It is a correct CRC operation in the “Neurogenesis:determination” GN that determines the implementation of the key event in the morphogenesis of each mechanoreceptor – the definition of a single sensory organ parental cell (SOPC), which is separated within a proneural cluster, the group of epidermal cells within imaginal disk (Furman, Bukharina, 2022). The parental cell differs from the surrounding ones by the content of proneural ASC proteins, coded for by the gene complex of the same name, achaete-scute complex or AS-C (Reeves, Posakony, 2005). An increased ASC content is the factor that determines the neural fate of a cell. By ensuring the development of both individual mechanoreceptors and their overall array, the so-called bristle pattern, the CRC regulates the production of these proteins to the level necessary for a cell to acquire an SOPC status (Furman, Bukharina, 2022).

Although the morphogenesis of mechanoreceptors has been long studied, it is unfortunately still far from an exhaustive description. It is only qualitatively characterized: the players in this process (genes and proteins) are known and the general concept of their interaction is formed; however, most of the quantitative parameters as well as a relative contribution of the involved genes have not been experimentally determined. Note that the scientists studying biological systems often encounter the situation of data incompleteness; here, mathematical and computer modeling is the tool allowing this problem to be resolved. A model with adequately selected parameters makes it possible not only to assess the current state of a system or an ongoing process, but also has a predictive value. Numerical experiments conducted with the help of mathematical models allow potential operation modes of a system to be examined, its future states to be forecasted, and its new functions to be predicted by changing parameters or adding new assumptions. In many cases, modeling is the only way to understand the processes taking place in a system when their characteristics cannot be directly measured in a biological experiment.

Modeling of the morphogenesis of mechanoreceptors at the stage of SOPC segregation from the cells of proneural cluster has been earlier attempted; however, the authors confined themselves to integrated characteristics and general schemes of intracellular and intercellular interactions of gene groups without (or with minimum) detailing of their composition and particular contributions of individual players (Marnellos, Mjolsness, 1998; Meir et al., 2002; Ghysen, Thomas, 2003; Hsu et al., 2006; Corson et al., 2017; Yasugu, Sato, 2022). Any integral concept of the mechanisms underlying the intracellular interactions in SOPC formation is still absent, as well as the quantitative characteristics for the content of ASC proteins critical for determining the neural fate of a cell are not determined and the degree of the influence of CRC components on the expression of AS-C genes is vague.

The goal of this work was to construct a mathematical model of CRC operation taking into account the roles of the constituent genes that would comprehensively describe the intracellular events in presumptive SOPC determining the dynamics of ASC content and to perform the computer experiments for verifying the model stability and its compliance with experimental data.

## Materials and methods

**Fig. 1.**
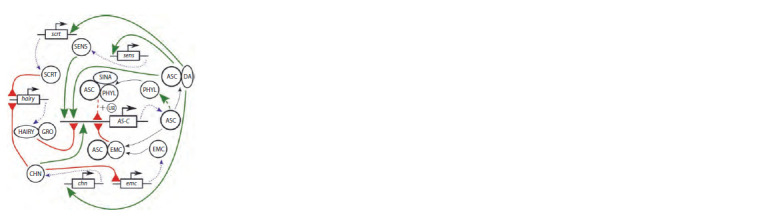
Scheme of the central regulatory circuit of the gene networks underlying the development of drosophila macrochaetes: AS-C, achaetescute gene complex; ASC, achaete-scute complex proteins; da, daughterless; gro, groucho; sens, senseless; emc, extramacrochaete; chn, charlatan; and scrt, scratch. Green arrows show activator effects (solid line, direct and dashed, mediated) and red arrows with chopped ends denote repressor effects (solid line, direct and dashed, mediated). The earlier published scheme (Golubyatnikov et al., 2015) has been updated by adding the ASC protein degradation system.

Object of modeling is the CRC (see Fig. 1 for the scheme). In addition to the AS-C proneural genes and the ASC proteins they code for, the circuit comprises the genes hairy, senseless (sens), charlatan (chn), scratch (scrt), phyllopod ( phyl ), and extramacrochaete (emc) and the corresponding proteins. The CRC also contains the proteins Daughterless (DA), Groucho (GRO), Ubiquitin (UB), and Seven-in-absentia (SINA). All components are connected with AS-C via activation–repression interactions.

The content of proneural ASC proteins in SOPC is determined via auto- and trans-regulation of AS-C gene activity. The activating autoregulation is implemented by the ASC/DS heterodimers and the repression, by ASC/EMC heterodimers. The trans-regulation of the CRC genes with an activating effect is performed by the Senseless and Charlatan proteins and with a negative effect, by the Hairy/GRO and ASC/EMC complexes (Cabrera, Alonso, 1991; Van Doren et al., 1992, 1994; Cabrera et al., 1994; Vaessin et al., 1994; Nolo et al., 2000; Escudero et al., 2005) (see Fig. 1).

Certain additional mechanisms make it possible to avoid the repressive effect of Hairy/GRO and ASC/EMC on AS-C. In particular, the activation of gene scratch by the ASC/DA heterodimers entails the repression of hairy transcriptional activity (Roark et al., 1995) and, as a consequence, an increase in the expression of AS-C. The activation of the chn gene represses the transcription of hairy and emc (Yamasaki et al., 2011) and leads to the same effect, that is, an increase in the AS-C expression (see Fig. 1).

Expression of the sens, scrt, and chn genes and, thus, the production of the corresponding proteins are regulated by the ASC/DA heterodimers, which initiate their transcription (Cabrera, Alonso, 1991; Vaessin et al., 1994; Nolo et al., 2000; Escudero et al., 2005) (see Fig. 1).

The CRC operation also requires the players involved in protein degradation, namely, ubiquitin (UB) and the E3 ubiquitin ligase Seven-in-absentia (SINA), as well as the adaptor protein Phyllopod (PHYL) (Pi et al., 2001; Chang et al., 2008).

Model. The proposed dynamical model of AS-C activity is described with a system of ordinary differential equations (1) (Bukharina et al., 2020):

**Formula.**
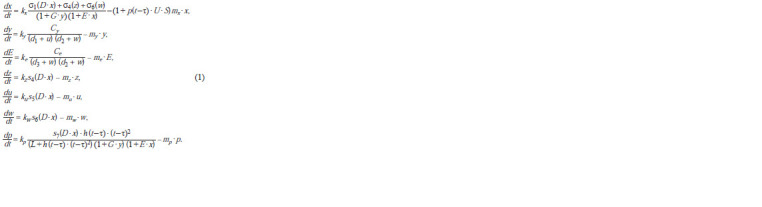
Formula

The variables in this system are the concentrations of the CRC proteins in the cell: x(t) is the content of ASC; y(t), of Hairy; E(t), of Extramacrochaete; z(t), u(t), w(t), and p(t), the concentrations of Senseless, Scratch, Charlatan, and Phyllopod, respectively.

To take into account the mutations of the genes that compose the CRC, the model contains non-negative coefficients kx , ky , ke , and so on reflecting the degrees of influence of the mutations on the synthesis of the corresponding proteins. The values of these coefficients do not exceed unity; k = 1 corresponds to the normal operation of a gene; and k = 0 denotes a complete inactivation of a gene and the absence of the corresponding protein.

Parameters x0, y0, z0, u0, w0, p0, and E0 denote the concentrations of the proteins ASC, Hairy, SENS, SCRT, CHN, PHYL, and EMC in the initial state of the CRC when the proneural cluster is already established, expression of all AS-C genes starts in all its cells, and all these cells still have equal neural potencies.

The values of parameters D, G, S, and U in system (1) are assumed constant since the concentrations of the corresponding proteins DA, GRO, SINA, and UB almost do not vary during the formation of parental cell. Parameters Cy , Ce , d1, d2, and d3 are assumed constant as well.

Positive coefficients mx , my , me , mz , mu , mw , and mp describe the degradation rates of the corresponding proteins.

The positive summand in the second equation of system (1) describes the negative feedbacks SCRT–Hairy and CHN–Hairy (see Fig. 1). The sigmoid functions σl , where l = 1, 4, 6 in the first equation of system (1), and the sigmoid functions si , where i = 4, 5, 6, 7 in the fourth–seventh equations of system (1), correspond to the positive feedbacks shown in Figure 1 with green arrows:

**Formula. 1.**
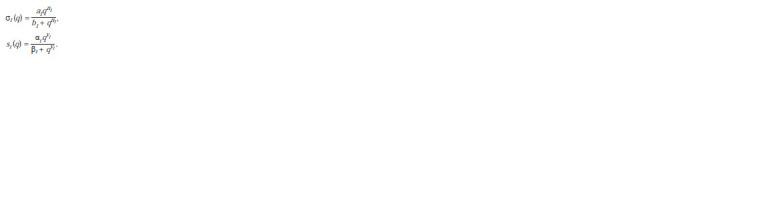
Formula 1

Here, αi , βi , νi and al , bl , nl are positive parameters, q ≥ 0 (Bukharina et al., 2015).

The model anticipates the choice of the CRC operation lifetime (T) and the moment (τ) when protein PHYL appears in the cell. The CRC functions until the cell starts to divide; hence, time T directly depends on τ: the later PHYL appears, the later the cell divides and the longer the CRC continues its operation. In the equation with delay, the function p(t0) is taken equal to 0 for 0 ≤ τ ≤ t.

Software. A special program complex based on the Shiny package has been designed for the numerical experiments with the CRC model described above and visualization of their results. The software makes it possible to elaborate interactive web applications with graphical user interface with the help of the R language (https://shiny.rstudio.com/).

The developed web application (https://gene-nets-simula tion.shinyapps.io/crc-asc-modeler/) allows the CRC operation modes to be simulated for different values of the parameters of system (1) and the results of these numerical experiments to be visualized as plots. Here, the parameters of the system are chosen in accordance with the results of biological experiments.

## Results and discussion

Let us consider the modeling results for different CRC operation modes.

Modeling of CRC operation in the presumptive parental cell of mechanoreceptor in the absence of any mutations in the constituent genes

**Fig. 2.**
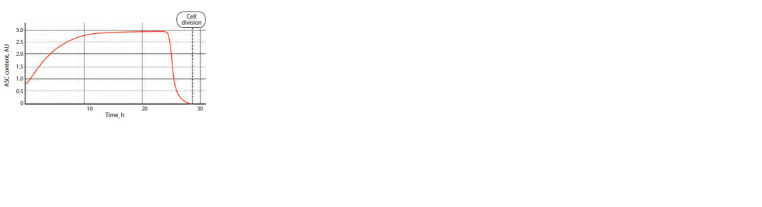
Dynamics of ASC protein content in the mechanoreceptor presumptive parent cell in the norm (AU, arbitrary units).

Figure 2 shows the results of computer simulation of CRC operation in the future SOPC in the norm (absence of any mutations in the CRC constituent genes). The parameters of system (1) were selected taking into account the available published experimental data (Reeves, Posakony, 2005; Chang et al., 2008; Giri et al., 2022):

**Formula. 2.**
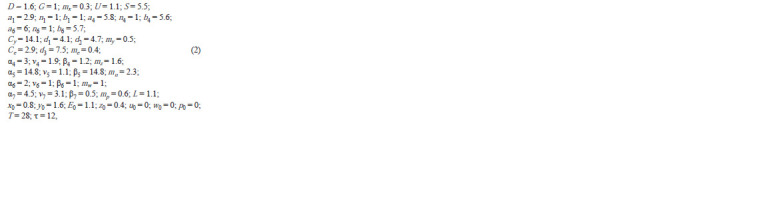
Formula 2

and coefficients k = 1 in all equations of system (1).

It is known that the SOPC determination for mechanoreceptors of different localizations takes different time (Cubas et al., 1991; Huang et al., 1991; Usui, Kimura, 1993). The time interval T = 28 h was selected as an interval close to the maximum necessary for determination of a neural cell fate (Huang et al., 1991). It is assumed that the CRC operation commences as early as the formation of proneural clusters 35–40 h before the puparium is formed when the expression of AS-C genes is first recorded (Cubas et al., 1991; Skeath, Carroll, 1991). The moment when proneural cluster is already formed, all its constituent cells display AS-C expression, and all of them still have equal neural potencies is regarded as the point zero.

The pattern of the changes in the content of ASC proteins in Figure 2 qualitatively matches the pattern observable in experiments (Reeves, Posakony, 2005; Chang et al., 2008). It is known that the content of ASC proteins gradually increases to reach a certain critical level after which the cell fate is unambiguously determined, namely, it becomes an SOPC. In the above-described numerical experiment, we got a smooth increase in the protein content over approximately 10 h to the level exceeding the initial one approximately 3.7-fold, that is, from 0.8 to 2.95.

Once the maximum is reached, the content of ASC proteins commences decreasing after a certain time interval to drop to almost zero value by the moment the SOPC starts dividing. This is determined by the switch-on of an additional regulatory mechanism associated with the degradation of ASC proteins (Chang et al., 2008). With the selected parameters, the model predicts that the ASC content commences to sharply decrease approximately in 15 h to reach the zero values during in 3 h.

It is important that the model excludes the possibility of any cyclic processes during the time interval limited by the moment of cell division, thereby demonstrating that the determination of a neural fate of the cell is irreversible. This also complies with the available published data (Reeves, Posakony, 2005; Chang et al., 2008).

According to different researchers, the SOPC segregation from proneural clusters for the mechanoreceptors of different localizations takes in the norm 9–12 to 28–30 h (Huang et al., 1991; Audibert et al., 2005; Kawamori et al., 2013). Note that the SOPC divisions for all mechanoreceptors are more or less synchronous and take place 0–3 h after pupation (Huang et al., 1991; Ayeni et al., 2016).

The first set of additional numerical experiments aimed at the testing of model stability to the change in time intervals required for the accumulation of ASC proteins in the amount necessary for the cell to achieve an SOPC status and to pass over to division (9 to 30 h). In this process, the value of parameter τ (the time moment when PHYL protein appears, which is critical for the transition of cell to division) was changed so that the values of parameter T (transition of SOPC to division) fall into the range of 9–30 h:

**Formula. 3.**
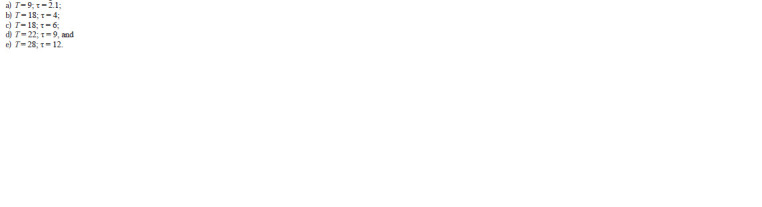
Formula 3

In additional experiments, the value of τ was taken to be 0 (that is, PHYL protein appeared simultaneously with ASC proteins) and parameter T was selected in an arbitrary manner to be 30 h or larger. The remaining parameters in these experiments remained constant and matched parameter set (2).

**Fig. 3.**
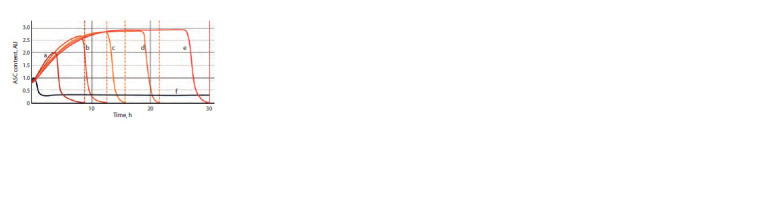
Dynamics of the content of ASC proteins in the presumptive parent cell of mechanoreceptor for different time parameters. (a–e) Parameter values are given in the text and (f ) τ = 0 and T = 30 h. Vertical dashed lines denote cell division.

Figure 3 shows the plots illustrating the dynamics of protein contents in mechanoreceptor parental cell at the selected time parameters. As is evident, the patterns of plots (a–e), shown by different tints of red, are similar to one another and the plot in Figire 2. The curves differ only in the duration of the phase when the ASC content is at its maximum level. Note that the shape of the curve is retained in the selected range of τ and the corresponding T values, thereby demonstrating that the proposed model of CRC operation is stable. For the case of τ = 0, which simulates the situation when PHYL (involved in the degradation of ASC proteins) appears without any delay, the shape of curve (f) in Figure 3, colored black, considerably differs from the remaining plots. The initial insignificant increase in the ASC content (not exceeding 16–17 % of the initial level) is followed by a decrease (to approximately half of the initial level) with subsequent plateau at a low level (although nonzero but insufficient for determining a cell as SOPC).

This result indirectly confirms the earlier assumption that a delayed appearance of the PHYL protein is the particular necessary condition for parent cell determination (Furman, Bukharina, 2022).

**Fig. 4.**
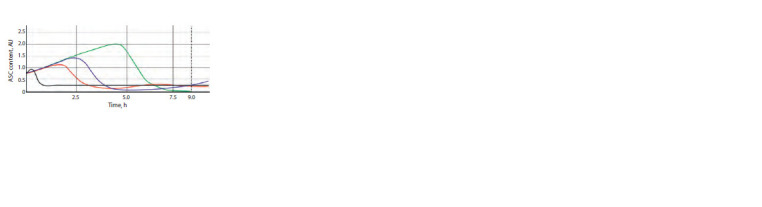
Evaluation of the minimum level of ASC protein content in the presumptive SOPC sufficient for the cell to acquire a neural status. See text for the values of time parameters.

This model makes it possible to gain the insight into the dynamics of ASC content in presumptive SOPC. By varying parameter τ, it is possible to assess what is the minimum necessary and sufficient excess amount of ASC proteins in a cell as compared with the content in the surrounding cells that ensure a neural status. Here, it is necessary to take into account the experimentally determined fact that this process requires at least 9 h (Huang et al., 1991; Audibert et al., 2005; Kawamori et al., 2013). Figure 4 shows the modeling results for the τ values of 0 h (black plot), 0.5 h (red plot), 1 h (blue plot), and 2.1 h (green plot).

The value of τ = 2.1 h is the first one when two conditions for cell transition to division are fulfilled: (1) the content of ASC proteins has dropped to zero and (2) time T amounts to approximately 9 h. This suggests that an approximately 2.5-fold increase in the ASC content in cell is already sufficient for the cell to follow a neural differentiation pattern.

The above data were obtained for the CRC operation in the norm. However, the model allows the relative contributions of CRC genes to its operation to be assessed as well by taking into account a mutation in each gene.

Modeling of CRC operation in the parental cell of mechanoreceptor in the presence of mutations in AS-C genes

As is known from experimental data, the mutations in achaetescute genes appear as the absence of part of mechanoreceptors and, in several cases, even all mechanoreceptors of the standard set (Agol, 1931; Dubinin, 1932; Cabrera et al., 1994; Roark et al., 1995; Pi et al., 2001; Escudero et al., 2005; Acar et al., 2006; Usui et al., 2008; Garcıa-Bellido, de Celis, 2009).

Several numerical experiments were performed to assess the effects of mutations in AS-C genes on the CRC operation. The following parameters of system (1) were used in these experiments:

**Formula. 4.**
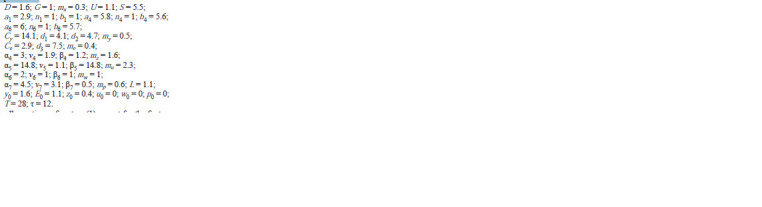
Formula 4

Table 1 lists the values of kxi and x0i . The value of parameter kxi varies from 0 (complete absence of protein) to 1 (protein content in the norm) and from a biological standpoint, reflects the degree of influence of a mutation in AS-C on the content of ASC proteins. The smaller the value of kxi , the lower is the content of the protein in the cell. Parameter x0i defines the initial content of ASC proteins. In the numerical experi- ments, x01 is assumed to be 0.8, which corresponds to the norm, kx1 = 1 (see Fig. 2). Coefficients kxi define a proportional decrease in the contents of proteins x0i according to equation x0i = x01· kxi .

**Table 1.**
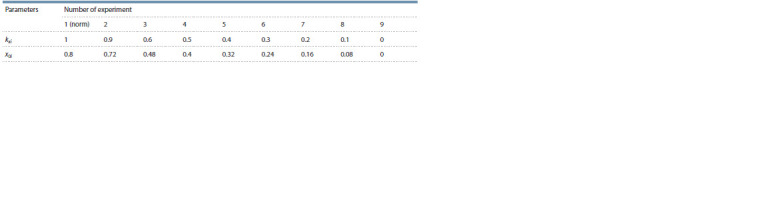
Values of parameters kxi and x0i in modeling the effect of mutations in ASC on the content of the corresponding proteins in presumptive SOPC

**Fig. 5.**
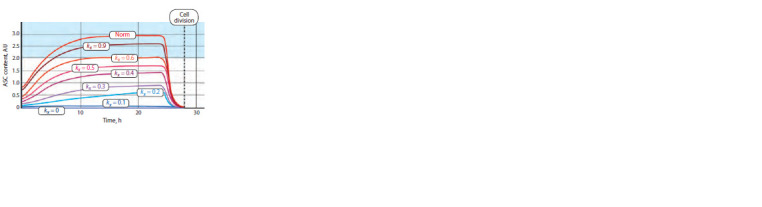
Dynamics of the content of ASC proteins in the mechanoreceptor parental cell in the presence of mutations in the achaete-scute gene complex. The region of ASC content at which SOPC determination is possible is colored turquoise.

Figure 5 shows the results of numerical experiments. The above-described data demonstrate that the determination of a cell as an SOPC in the absence of mutations in the CRC genes becomes possible when the ASC content increases at least 2.5-fold as compared with the initial value (see Fig. 4). Thus, it is possible to assess the minimum kxi value when this condition is met. The range of the content of ASC proteins permitting the determination of SOPC is colored turquoise. The plots showing the content of ASC proteins corresponds to the kxi values at which the possibility of cell determination as an SOPC is retained.

The necessary level of the content of ASC proteins is achieved at kxi ≥ 0.6. The value of kx3 = 0.6 corresponds to a decrease in the content by 40 % relative to the initial values of the norm. From a biological standpoint, this means that a decrease in the ASC content in the cell by >40 % prohibits its differentiation according to a neural pathway and, consequently, entails the absence of mechanoreceptor.

Modeling of CRC operation in the presumptive SOPC in the presence of mutations in constituent genes

The CRC components are united via the intracellular system of positive and negative feedbacks (see Fig. 1), which strictly regulates the production and degradation of ASC proteins. Correspondingly, the mutations in each gene must influence the content of the corresponding proteins in the cell and have a certain phenotypic effect. Indeed, experiments have shown that the mutations of CRC genes appear as variations in the canonical architecture of bristle pattern, namely, changes in the number and/or positions of mechanoreceptors. The considered model that takes into account the mutational changes in CRC genes allows the degree and character of their effects on the dynamics of ASC content to be assessed. In the numerical experiments, coefficients ky (for hairy), ke (for emc), kz (for sens), ku (for scrt), kw (for chn), and kp (for phyl) were assumed to be zero, which corresponds to a complete absence of the corresponding proteins.

Several parameters remained constant:

**Formula. 5.**
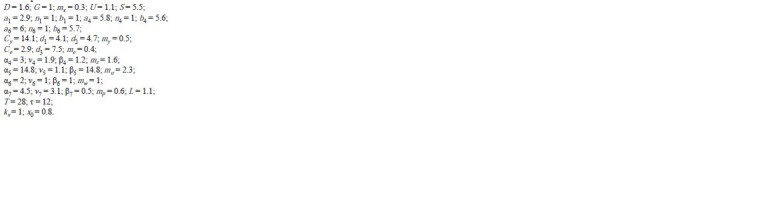
Formula 5

The changing parameters are listed in Table 2: the k values of 0 or 1 mean the presence or absence of a mutation in a gene and parameters y0, z0, u0, w0, p0, and E0 specify the initial contents of the proteins Hairy, SENS, SCRT, CHN, PHYL, and EMC, respectively

**Table 2.**
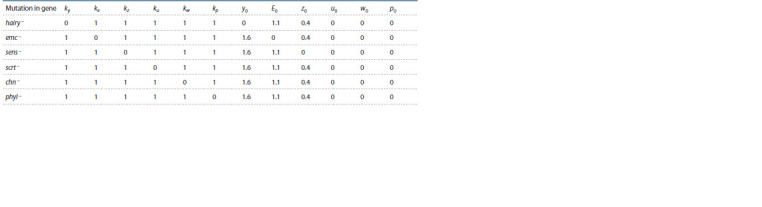
Values of changing parameters in modeling the effect of mutations in CRC genes on the content of ASC proteins

**Fig. 6.**
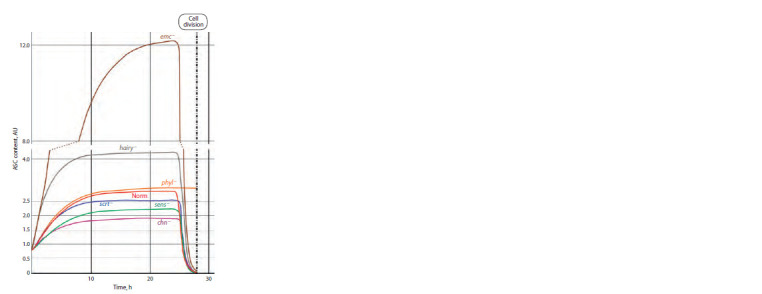
Dynamics of the content of ASC proteins in the mechanoreceptor presumptive parent cell in the presence of mutations in CRC genes.

Figure 6 shows the results of numerical experiments. A comparison of the shapes of the plots shown in Figure 6 reveals a certain hierarchy of the CRC genes in their effects on the content of ASC proteins. This is reflected in the range of deviations from the plot that characterizes the dynamics of these proteins in the norm (in the absence of any mutations in all genes of the CRC). The larger the deviation, the stronger is the effect of an individual gene.

The emc (emc –) and hairy (hairy –) genes display the strongest effects because the mutations in these genes cause a considerable upward deviation of the ASC level from the normal characteristics. This is a biologically justified result since the EMC and Hairy proteins repress AS-C (Moscoso del Prado, Garcia-Bellido, 1984) so that the removal of this repression must appear as an increase in ASC content. A phenotypic manifestation of mutations consists in the development of additional mechanoreceptors (Ingham et al., 1985; de Celis et al., 1991). Presumably, a concurrent sharp and rapid increase in the ASC content in the cells of proneural cluster causes mistuning of intercellular interactions mediated by signaling pathways and the formation of several SOPCs in the proneural cluster rather than a single SOPC as in the norm.

The mutation in chn (chn –) appears as a noticeable decrease in the ASC level (the corresponding curve lies below the curve for the norm). The effect is associated with the fact that the mutation in this gene causes the absence of the corresponding protein, which directly activates the AS-C genes and represses the emc and hairy genes (Escudero et al., 2005; Yamasaki et al., 2011). Correspondingly, the production of ASC proteins cannot reach the required values.

The mutations in genes sens (sens –) and scrt (scrt –) cause a less pronounced increase in the level of proteins, which also agrees with the known data on the functions of these genes in the CRC system and the manifestations of mutations in these genes. The SENS protein is known as a coactivator of AS-C activity and, consequently, the mutation will somewhat decrease the ASC production. The SCRT protein represses the hairy gene, thereby potentially increasing the ASC level, which, nonetheless, fails to reach the normal values because of the effects of other direct repressors of AS-C gene activity (Roark et al., 1995; Nolo et al., 2000) (see Fig. 1).

In the case of a mutation in the phyl gene (phyl –), the ASC level expectedly remains on the reached plateau because the PHYL protein, responsible for its degradation, is not produced in this case (Chang et al., 2008). Thus, SOPC cannot transit to division and the phenotypic effect must appear as the absence of mechanoreceptor at its regular position. This conclusion is confirmed by experimental data (Pi et al., 2001).

## Conclusion

The decades of the research into the system underlying the formation of bristle pattern on the head and body of drosophila have yielded a tremendous array of data giving the insight into individual mechanisms forming the basis for the function of this system. However, particular details of the morphogenesis of mechanoreceptor are still rather vague.

We have earlier demonstrated that the development of an individual mechanoreceptor and the overall bristle pattern are controlled by the central regulatory circuit, which determines the expression of AS-C genes and production of the corresponding proteins in the parental cell. A mathematical model of the CRC operation was elaborated taking into account all identified CRC components and the relations between them. This model allowed us to advance from a purely qualitative description of the system controlling the content of ASC proteins and to succeed in clarification of its certain quantitative characteristics unknown earlier

In particular, our numerical experiments suggest that the cell is determined as an SOPC when the ASC content increases approximately 2.5-fold relative to the initial level in the cells of proneural cluster. Individual elements of the circuit have different effects on the content of ASC proteins in the presumptive cell of mechanoreceptor. AS-C, the key CRC component, and the mutations that decrease the ASC content by more than 40 % have the most significant effect and cause the prohibition of SOPC segregation. As for the mutations in the remaining genes of the circuit, they change the level of ASC proteins to different degrees, with the most pronounced effects of mutations in the emc and hairy genes.

Thus, the model demonstrates that the CRC as a system is sensitive to changes in internal interactions and its robust operation, providing a certain dynamics of the level of ASC proteins, requires a concerted work of all components constituting the regulatory circuit. The model predictions are appropriate for experimental verification.

## Conflict of interest

The authors declare no conflict of interest.
